# Presence of *Dioctophyme renale* in domestic and wild cycles in the Brazilian Cerrado

**DOI:** 10.1590/S1984-29612025016

**Published:** 2025-03-31

**Authors:** Zara Mariana de Assis-Silva, Lizandra Fernandes-Silva, Iago de Sá Moraes, Bruna Samara Alves-Ribeiro, Reiner Silveira de Moraes, Doughlas Regalin, Ísis Assis Braga, Klaus Casaro Saturnino, Dirceu Guilherme de Souza Ramos

**Affiliations:** 1 Programa de Pós-graduação em Biociência Animal, Unidade Acadêmica de Ciências Agrárias, Universidade Federal de Jataí –UFJ, Jataí, GO, Brasil; 2 Laboratório de Parasitologia e Análises Clínicas Veterinária, Instituto de Ciências Agrárias, Universidade Federal de Jataí – UFJ, Jataí, GO, Brasil; 3 Laboratório de Anatomia Patológica Veterinária, Instituto de Ciências Agrárias, Universidade Federal de Jataí – UFJ, Jataí, GO, Brasil; 4 Departamento de Clínica Veterinária, Faculdade de Medicina Veterinária e Zootecnia, Universidade Estadual Paulista Júlio de Mesquita Filho – UNESP, Botucatu, SP, Brasil; 5 Hospital Veterinário, Instituto de Ciências Agrárias, Universidade Federal de Jataí – UFJ, Jataí, GO, Brasil

**Keywords:** Canides, fragmentation, micro-region, zoonosis, Canídeos, fragmentação, microrregião, zoonose

## Abstract

Despite several records of *Dioctophyme renale* in domestic hosts (mainly dogs) and wild animals in Brazil, there are few studies related to the biology of the parasite and epidemiology of the parasitosis in the country. This also applies to the interactions and scratches in different locales. The aim of this study was to describe *D. renale* occurrence in 12 animals at an interface of the domestic-wild cycle and to detail two specific cases of these, one domestic and one wild canine (*Chrysocyon brachyurus*) from the same microregion of the Brazilian Cerrado. The difficulty in diagnosing dioctophimosis is related to nonspecific clinical signs. Over the last five years, 12 cases of *D. renale* have been reported in domestic and wild canids, two of which are described in full in this study. The expansion of livestock farming and urbanization of biomes, fragmentation of floral areas, and destruction of natural areas have increased the proximity between domestic and wild animals, and consequently, the occurrence of infectious and parasitic diseases. Its probability of occurrence in humans represents a public health risk.

## Resumo

Apesar de diversos registros de *Dioctophyme renale* em hospedeiros domésticos (principalmente cães) e animais silvestres no Brasil, existem poucos estudos sobre a biologia do parasito e a epidemiologia da parasitose no país. Isso também se aplica às interações e variações regionais. O objetivo deste estudo foi descrever a ocorrência de *D. renale* em 12 animais em uma interface do ciclo doméstico-selvagem e detalhar dois casos específicos destes, um canino doméstico e um selvagem (*Chrysocyon brachyurus*) da mesma microrregião do Cerrado brasileiro. A dificuldade no diagnóstico da dioctofimose está relacionada aos sinais clínicos inespecíficos. Nos últimos cinco anos, 12 casos de dioctofimose foram relatados em canídeos domésticos e selvagens, dos quais dois são descritos na íntegra neste estudo. A expansão da pecuária e a urbanização dos biomas, a fragmentação das áreas florais e a destruição das áreas naturais aumentaram a proximidade entre os animais domésticos e silvestres e, consequentemente, a ocorrência de doenças infecciosas e parasitárias. Sua probabilidade de ocorrência em humanos representa um risco à saúde pública.

*Dioctophyme renale* Goeze 1782 (Enoplida: Dioctophymatidae) is blood red in color. Males measure 15–45 cm in length and 3–4 mm in diameter, whereas females measure 20–100 cm in length and 5-12 mm in diameter (Russo et al., 2022). Dioctophymosis is a cosmopolitan disease that affects domestic and wild carnivores, and the infections are mainly related to the ingestion of intermediate and paratenic hosts (fish and frogs) (Measures & Anderson, 1985). The eggs are eliminated in the urine of the definitive host, and their development occurs in aquatic environments (Pedrassani et al., 2017).

In Canidae, infection generally occurs in the right kidney; however, *D. renale* can be found in the subcutaneous, mediastinal, peritoneal, and internal organ tissues when it causes an ectopic form of the disease (Ilić et al., 2023). Clinical manifestations, such as renal colic, dysuria, hematuria, pyuria, fever, anorexia, anemia, abdominal distension, and convulsions, are not specific. Although not considered an important zoonosis, sporadic reports have been published in humans (Yang et al., 2019). Since 2010, they have been carried out in Brazil and in various biomes such as Pampa (Trindade et al., 2018), Cerrado (Vulcani et al., 2015), and Atlantic Rainforest (Pedrassani et al., 2017) in domestic and wild animals. In the southwestern microregion of the state of Goiás, the most recent occurrence was in maned wolf in 2015 (Vulcani et al., 2015). In addition to these, there is a recent report in the Bolivian Pantanal, in San Matías (Tancredi et al., 2021).

By considering the complexity of the biological cycle of *D. renale* and the difficulty of diagnosis (being a necroscopic finding in most cases), the comprehension of epidemiological aspects of the transmission and maintenance of parasitosis in different biomes, as well as the interactions and risks in different locations, is not yet fully disseminated. Although various records document occurrences in the literature, routine clinical urine analyses and ultrasounds are not frequently conducted, likely leading to underreported prevalence rates. Incidental findings of *D. renale* have been reported in South America through routine surgical procedures such as ovariohysterectomy and orchiectomy (Greer et al., 2021). In this context, the present study aimed to report the circulation of *D. renale* in domestic and wild animals in the same microregion of the Brazilian Cerrado. We recorded 12 cases of *D. renale* in wild and domestic canids, including two full reports in the southwestern microregion of Goiás, which until had no records of this parasite in domestic animals.

A survey of animals with diocthymosis was conducted at the southwest of the state of Goiás for 2020-2024 in the municipalities of Santa Rita do Araguaia (17°19'33”S, 53°12'18”W), Mineiros (17°34′43″S, 52°32′33″W), Serranópolis (18°17′38″S, 51°58′10″W) and Jataí (17°52′33″S, 51°43′17″W), which is an area of the Brazilian Cerrado, comprising 18 municipalities covering 56,112.15 km^2^ and has a population of 558,560 inhabitants. Over the last five years, 12 cases of *D. renale* have been recorded in domestic and wild canids dogs, nine in domestic dogs (n_Santa Rita do Araguaia_=1; n_Mineiros_=1; n_Serranópolis_=2; n_Jataí_=5) and three cases in maned wolves (*Chrysocyon brachyurus*) (n_Mineiros_=1; n_Serranópolis_=1; n_Jataí_=1). The domestic animals were brought by their owners to the Veterinary Hospital of the Federal University of Jataí (UFJ), while the wild animals were sent by the Environmental Departments of the aforementioned municipalities, after captures in anthropic rural areas and the helminths collected after the surgical procedure were sent to the Laboratory of Parasitology and Veterinary Clinical Analysis at UFJ (reference laboratory for identifying helminths in the region) for identification and confirmation of the diagnosis.

In a chronological line, the cases began to occur in 2020, when no frequent occurrences of dioctophimosis in the clinical routine of the region were reported. Two cases in domestic dogs from Jataí in 2020, where the animals were referred for surgical care at the UFJ Veterinary Hospital, after initial care in private veterinary clinics. In these cases, our research team did not have access to all the data on the conduct, and we received only the helminths that are deposited in the UFJ Parasitology teaching collection. In the same year, a *C. brachyurus* found in Serranópolis was diagnosed with dioctophimosis, monitored by our research team and described in its entirety. This was the first year with three records of the disease in the region, where only sporadic cases were reported. In 2021, another domestic dog from Jataí was diagnosed with the parasitosis, an animal that we monitored completely and described in this study. Also in 2021, two more cases were recorded in domestic dogs (in Jataí and Santa Rita do Araguaia) and one case of *C. brachyurus* in Mineiros. In 2022, one case was recorded in a domestic dog in Serranópolis and one in *C. brachyurus* in Jataí. In 2023, one more case was recorded in a domestic dog in Serranópolis and one in Mineiros. Lastly, in 2024, a case was recorded by our team in a domestic dog in Jataí. The cases that were not fully reported were cases not fully monitored by our research team, generally attended by Veterinary Medicine residents at the UFJ Veterinary Hospital without complete collection or recording of case information, or even in emergency care, with the identification of helminths after referral to the Laboratory of Parasitology and Veterinary Clinical Analysis at UFJ, which were identified and deposited in the Parasitology teaching collection together with information on the year and hosts of origin.

Of these 12 cases, we report two cases in full, one domestic and one wild canine (*C. brachyurus*) underwent clinical evaluation and underwent ultrasound examination, urinalysis and, when applicable, exploratory laparotomy, with removal of the injured tissues along with the parasites, and unilateral nephrectomy.

A maned wolf (*C. brachyurus*), female, found on a farm in the municipality of Serranópolis, was treated at the UFJ Veterinary Hospital. The canid was mildly dehydrated, had pale mucous membranes, was thin, had a tense abdomen, and showed abdominal discomfort. There were old injuries, such as an amputated tail, and recent injuries with myiasis in the right ear.

**Figure 1:**
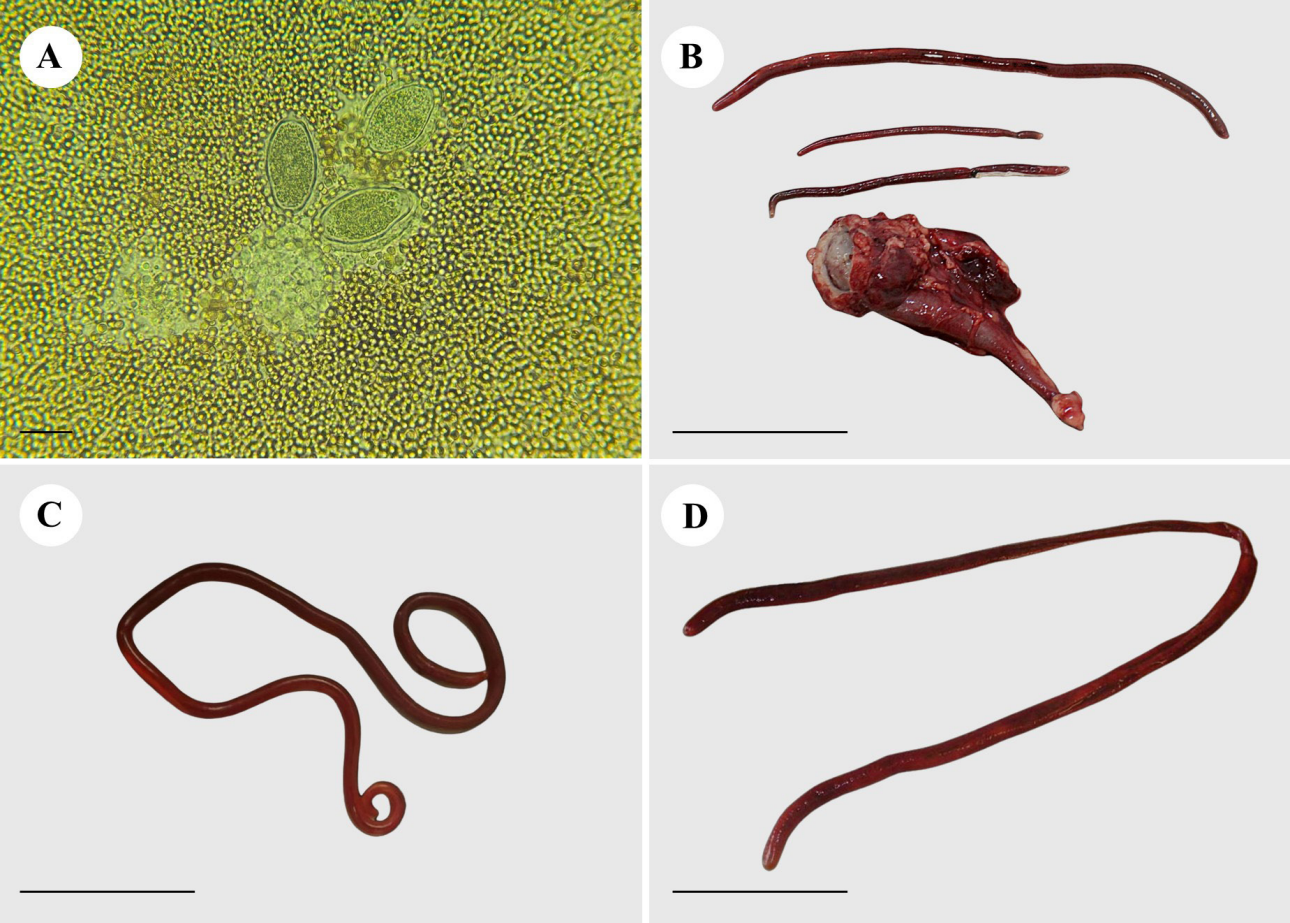
*Dioctophyme renale* found in maned wolf (*Chrysocyon brachyurus*) and domestic dogs (*Canis familiaris*) in the same microregion of the Brazilian Cerrado. (A) *Dioctophyme renale* eggs in the maned wolf’s urine sediment (scale: 100 μm); (B) Specimens found in the wolf's right kidney (scale: 5 cm); (C) Male found in the abdominal cavity of a maned-wolf (scale: 5 cm); (D) Female found in the right kidney of a domestic dog (scale: 5 cm).

A blood count revealed normocytic normochromic anemia, mild thrombocytopenia, leukocytosis due to neutrophilia, with a left shift, and the presence of *Anaplasma* spp. Urinalysis revealed traces of proteins, ketone bodies, and a marked presence of hemoglobin. During urinary sedimentation, a marked number of pyocytes, red blood cells, and bacterial microbiota were observed along with hyaline and granular casts, triple phosphate crystals, and *D. renale* eggs (Figure1A). The presence of *D. renale* in the right kidney was confirmed using abdominal ultrasonography.

Nephrectomy was indicated based on clinical and imaging findings (Eiras et al., 2021). During surgery, a female specimen (20 cm in length) was found in the abdominal cavity. Three parasites were found after removal and dissection of the kidney, in addition to the renal parenchyma being completely destroyed (Figure 1B and 1C), and presented the diagnostic characteristics of *D. renale* (Russo et al., 2022).

In a second case, a canine, female, mixed breed, five years old, from the city of Jataí, Goiás, was treated at the UFJ Veterinary Hospital with a history of hyporexia, daily episodes of vomiting, oliguria and presence of melena with an increased abdominal volume located on suspicion of being run over. The animal had free access to the street and had an unknown history of immunoprophylaxis and antiparasitic drugs.

The animal appeared apathetic, with intense dehydration (12%), enophthalmos, loss of skin elasticity, weak pulse, congested mucous membranes, petechiae distributed throughout the body, and an abdominal hematoma. Blood tests revealed anemia, severe thrombocytopenia, and leukopenia. Enzyme biochemical measurements revealed severe renal with creatinine (4.6 mg/dL) and urea (460 mg/dL) levels above their reference values, and hepatic deficiency. In addition to immediate fluid therapy with Ringer's lactate (5 ml/kg/h) administered intravenously, outpatient treatment was initiated with doxycycline (10 mg/kg BID), ondansetron (0.22 mg/kg TID), ranitidine (2 mg/kg), maropitant citrate (1 mg/kg SID), a vitamin complex with ferrous sulfate (1 tablet SID), and dietary supplementation.

During the ultrasound examination, the usual topography was observed in both kidneys with defined contours and regular shapes; however, the examination indicated the presence of hydronephrosis and bilateral renomegaly. The bladder did not have a usual topography with an anechoic content and a high amount of suspended sediment, suggesting cystitis or obstruction.

After seven days, the animal died. Upon necroscopic examination, hepatomegaly was observed, showing a central lobe pattern, with a “nutmeg” appearance. There was a moderate amount of foam in the trachea and a slightly reddish lung with abundant serous discharge upon cutting. The left kidney was larger than the right kidney with dilation of the renal pelvis. A nematode, *D. renale*, was found inside the right kidney, which had been destroyed and contained bloody fluid (Figure 1D). The stomach had diffuse red mucosa (gastritis), and the intestine had bloody stools. The blood content was observed in the bladder.

The diagnosis of *D. renale* is made difficult by the non-specificity of the clinical signs, with unilateral manifestations tending to hypertrophy of the contralateral, unaffected kidney and, most of the cases in the past were from necropsy findings (Ferreira et al., 2010). Oliveira et al. (2021) described renal interstitial fibrosis with hypotrophy of the medullary cortical region, which is consistent with the lesions observed in the two cases in this study. In females, immunosuppression owing to frequent pregnancies contributes to higher rates of parasitism (Pedrassani et al., 2017). In both cases fully described in this study, the animals were female but without proof of gestational history.

Russo et al. (2022) recently reviewed the topic and concluded that animals affected by *D. renale* are generally asymptomatic, with no significant alterations in hematological and biochemical tests. The authors highlight that symptomatic cases often exhibit urinary lesions, along with changes in urea and creatinine levels. These findings align with those observed in the domestic dog described in our study, which presented oliguria and elevated urea and creatinine levels. In this case, the additional alterations were likely a consequence of the rapid progression of renal lesions. In *C. brachyurus*, leukocytosis was observed, but is more commonly associated with ectopic dioctophimosis, not observed in the animal from our study. Instead, we attributed the other conditions present, such as anaplasmosis and bodily lesions, and dioctophimosis appeared to be an incidental finding. The diagnosis was made through urinalysis, where *D. renale* eggs were identified, a characteristic feature frequently described in the literature (Russo et al., 2022).

The ecoepidemiology of dioctophymosis is associated with regions with high water levels where intermediate and/or paratenic hosts are abundant (Russo et al., 2022). The habit of drinking water from canals or eating fish or anurans has been identified as a risk factor for domestic and/or wild animals in places with a high prevalence (Mascarenhas et al., 2019). Collaboration between paratenic hosts, although not essential for the completion of the cycle, increases the spread of infection and contributes to its maintenance in the environment (Russo et al., 2022). In summary, the eggs produced by mature females in the kidneys of definitive mammalian hosts are excreted in the urine, subsequently ingested by the first intermediate host, the freshwater oligochaete *Lumbriculus variegatus*, where the eggs develop into the third-stage larval phase, and mammalian definitive hosts, typically carnivores, can become infected by drinking water containing the infected intermediate host or by consuming paratenic hosts (Eiras et al., 2021).

The Southwest microregion of the Brazilian state of Goiás is located in the Cerrado biome and is represented by forest, savannah, and grassland vegetation, a humid temperate climate, and well-defined rainy and dry seasons. The average temperature varies from 18 to 32ºC and its hydrography is represented by the Paranaíba river basin and its tributaries (Souza, 2014). This microregion (where the municipalities in this study are located), has the necessary characteristics for the development of the biological cycle, both in terms of climate and the biodiversity of definitive, intermediate, and paratenic hosts.

In addition to the maned wolf, *D. renale* has been recorded in coatis (*Nasua nasua*), otters (*Lutra longicaudis*), sloths (*Choloeupus didactylus*), capuchin monkeys (*Sapajus apella*) (Eiras et al., 2021), geoffroy's cat (*Leopardus geoffroyi*) (Trindade et al., 2018), lesser grison (*Galictis cuja*) (Pesenti et al., 2012), and neotropical river otter (*Lontra longicaudis)* (Echenique et al., 2018). The expansion of agriculture, the main production of the study site, and urbanization have increased the anthropization of biomes, the fragmentation of forest areas, and the destruction of natural areas. These factors increase the proximity between wild and domestic animals (Soler et al., 2005) and, consequently, the occurrence of infectious and parasitic diseases is shared, as presented in this study.

The prevalence of dioctophymosis varies according to the region assessed, ranging from 0.49% to 100% (Eiras et al., 2021; Tancredi et al., 2021), with reports in some Brazilian states, such as Pará (Miranda et al., 1992), Mato Grosso (Pizzinatto et al., 2019) Minais Gerais (Costa & Lima, 1988), Espírito Santo (Barros, 1971), Rio de Janeiro (Duarte, 1981), São Paulo (Torres et al., 2001), Santa Catarina (Pedrassani & Nascimento, 2015), Rio Grande do Sul (Brun et al., 2002). In humans, dioctophimosis has been confirmed in many countries such as Austria, Bulgaria, Chile, Indonesia, Iran, India, Japan, Greece, Korea, Thailand and Yugoslavia, and its presentation varies from asymptomatic, nonspecific clinical signs, such as lower back pain and urinary retention, sometimes mimicking cancer or neoplasms, with cases rarely leading to death (Yang et al., 2019; Eiras et al., 2021). Until 2020, only a single record of *C. brachyurus* had been documented in the study region by Vulcani et al. (2015), and reports of the parasite in domestic animals were extremely rare. Between 2010 and 2019, only two cases were recorded at the UFJ Veterinary Hospital—a reference center within a 400 km radius (unpublished data). However, the sudden surge in dioctophimosis cases, reaching endemic levels, raised two critical hypotheses that warrant further investigation in the coming years and serve as the basis for this study: Was dioctophimosis previously underdiagnosed, or has there been an increase in the parasite’s abundance in the region? The concern of this study was that domestic/wild occurrence in a fragmented hotspot biome may pose a risk to humans, as fishing is frequent in this region.

Mascarenhas et al. (2019) report the presence of third-stage larvae of *D. renale* in fish from the southern region of Brazil, when they discuss the role of paratenic hosts in the transmission and maintenance of parasitosis in domestic and wild animals. According to Yang et al. (2019), the consumption of raw fish, frogs, and contaminated water are suspected sources of infection. The presence of parasitized animals in the region should be considered a public health concern, as the domestic and wild circulation of the parasite increases the probability of occurrence in humans, and the proximity of the two niches is becoming evident.
